# Compassionate drug (mis)use during pandemics: lessons for COVID-19 from 2009

**DOI:** 10.1186/s12916-020-01732-5

**Published:** 2020-08-21

**Authors:** Amanda M. Rojek, Genevieve E. Martin, Peter W. Horby

**Affiliations:** 1 Epidemic Diseases Research Group Oxford (ERGO), Centre for Tropical Medicine and Global Health, Nuffield Department of Medicine, University of Oxford, Old Road Campus, Roosevelt Drive, Oxford, OX3 7FZ UK; 2 Emergency Department, The Royal Melbourne Hospital, Melbourne, Victoria Australia; 3 Centre for Integrated Critical Care, University of Melbourne, Melbourne, Victoria Australia; 4 Department of Allergy, Immunology and Respiratory Medicine, The Alfred Hospital, Melbourne, Victoria Australia

**Keywords:** Pandemic, Clinical trial, Influenza, H1N1, Systematic review

## Abstract

**Background:**

New emerging infections have no known treatment. Assessing potential drugs for safety and efficacy enables clinicians to make evidence-based treatment decisions and contributes to overall outbreak control. However, it is difficult to launch clinical trials in the unpredictable environment of an outbreak. We conducted a bibliometric systematic review for the 2009 influenza pandemic to determine the speed and quality of evidence generation for treatments. This informs approaches to high-quality evidence generation in this and future pandemics.

**Methods:**

We searched PubMed for all clinical data (including clinical trial, observational and case series) describing treatment for patients with influenza A(H1N1)pdm09 and ClinicalTrials.gov for research that aimed to enrol patients with the disease.

**Results:**

Thirty-three thousand eight hundred sixty-nine treatment courses for patients hospitalised with A(H1N1)pdm09 were detailed in 160 publications. Most were retrospective observational studies or case series. Five hundred ninety-two patients received treatment (or placebo) as participants in a registered interventional clinical trial with results publicly available. None of these registered trial results was available during the timeframe of the pandemic, and the median date of publication was 213 days after the Public Health Emergency of International Concern ended.

**Conclusion:**

Patients were frequently treated for pandemic influenza with drugs not registered for this indication, but rarely under circumstances of high-quality data capture. The result was a reliance on use under compassionate circumstances, resulting in continued uncertainty regarding the potential benefits and harms of anti-viral treatment. Rapid scaling of clinical trials is critical for generating a quality evidence base during pandemics.

## Background

Viral pandemics constitute a major threat to global health security. Future influenza pandemics are considered likely. In the past 20 years, we have also seen the emergence of zoonotic human respiratory coronaviruses with pandemic potential. These have been severe acute respiratory virus (SARS; caused by SARS-CoV-1), Middle East respiratory syndrome (MERS) and, currently, coronavirus disease 2019 (COVID-19) (caused by SARS-CoV-2) [1]. A study of the 2009 H1N1 strain influenza A (A(H1N1)pdm09) pandemic, the largest respiratory viral outbreak in recent years, can provide insights into the research processes during a pandemic, with the aim of improving these for other outbreaks, including COVID-19.

One important element of pandemic mitigation is prophylaxis and treatment of patients. For emerging viral infections, antiviral therapies are a key medical countermeasure because vaccine production takes months or years, whereas effective antiviral medications may already exist. For COVID-19, the potential of several existing medications (including remdesivir, lopinavir/ritonavir, hydroxychloroquine and tocilizumab) is of interest. During the influenza A(H1N1)pdm09 pandemic, there was interest in neuraminidase inhibitors (NAIs) as anti-influenza agents, although adequate safety and efficacy data supporting their use were lacking. This evidence has now substantially strengthened [2]; however, much of this was generated after the pandemic. There has been no quantitative assessment of the volume and quality of information that is produced regarding treatments during the pandemic period. This data is important, because it represents what is available to clinicians making treatment decisions for patients under conditions of significant uncertainty [3], and during surging patient numbers [4].

The objective of this systematic review is to investigate how data for drug treatments of A(H1N1)pdm09 accrued during the pandemic (detailed objectives listed in Table 1). This review is not only limited to completed clinical trials, but also includes registered trials which were not completed, and reports of treatment outside a formal trial setting (case studies or series and observational studies). These are included as they may represent both the best quality evidence available at the time and also opportunities lost to gather high-quality evidence.

**Table 1:** Detailed objectives and rationale with respect to evidence generation during a pandemic

Aim	Reason
Quantify the volume of data that described patient treatment, stratified by research type	The volume of research gives an indication of the scale of the response mounted. Comparisons of different types of research (clinical trial, hypothesis-driven observational study, case series) describe the quality of evidence available
Document the time taken to initiate and complete this clinical research and compare this to the outbreak epidemiology	The faster that clinical research is commenced, the greater the pool of potential participants, and the greater the likelihood of enrolling a sufficient sample size and completing within the timeframe of the outbreak
Document the time taken to submit and publish this research and compare this to the outbreak epidemiology	Research can only influence patient treatment in the current outbreak by providing enhanced evidence if it is available to clinicians treating patients within the time-frame of the outbreak
Describe the extent to which manuscripts report key clinical parameters, including those needed for stratification of treatment effect (the age of patients, the pregnancy status of patients) or indicate the quality of reporting of treatment effect (adverse events due to treatment)	We have selected key parameters for assessment that would be necessary to know in order to evaluate a treatment effect.
Describe the outcomes of clinical research that was prospectively registered	This expands discussion around limits to conducting high-quality research as we can comment on the proportion of planned research that was able to complete.

## Methods

We conducted a systematic search to identify patients treated for A(H1N1)pdm09 during the pandemic. We searched two types of evidence: peer-reviewed publications and clinical trial registration records. An experienced librarian advised on the search strategy. We prospectively registered the review (PROSPERO database record CRD42016039549). Details of compliance to MOOSE and PRISMA guidelines are found in Additional file 1: appendix 1.

### Published literature search

We searched the PubMed database according to the search strategy found in Additional file 1: appendix 2. To capture information on how many patients received treatment outside of a trial, we included case studies, case series and observational research in addition to interventional research. The single exception was to limit descriptions of the use of oseltamivir to publications with ten or more patients, because case reports were abundant. We included research that described hospitalised patients and reported acute clinical outcomes (defined as the length of hospitalisation, intensive care admission or length of stay, medical complication, requirement for mechanical ventilation or mortality). We included patients with only laboratory-confirmed disease. While A(H1N1)pdm09 was the prevailing strain during the outbreak, inconsistencies in defining probable cases between papers meant a consistent inclusion method was not possible. We included papers only if enrolment opened between April 1, 2009 (when the virus strain was first identified), and was completed by August 10, 2010 (the declaration of the end of the Public Health Emergency of International Concern [PHEIC]), by the World Health Organization (WHO). This limitation was necessary to differentiate research conducted specifically for the pandemic, compared with routine seasonal influenza reporting once A(H1N1)pdm09 became a seasonal strain. These criteria did not apply for clinical trials (where there could be no confusion with seasonal reporting).

We excluded papers if the description of treatment was not quantifiable or the treatment name was absent (including use of the general term ‘antiviral therapy’). We defined treatment as pathogen-directed therapy (e.g. antivirals) or host-directed therapy where there was a specific indication for A(H1N1)pdm09. We therefore excluded descriptions of standard intensive care interventions including corticosteroids and extracorporeal membrane oxygenation. When a single patient cohort (same sample size, enrolment period, author(s) and study location) was presented in more than one paper, duplicates were excluded. We excluded languages other than English.

### Clinical trial registry search

We undertook two clinical trial registry searches. The purpose of the first was to examine research that was planned in response to the pandemic. ClinicalTrials.gov was searched using the condition ‘H1N1’ and dates were restricted to limit to registration dates following the onset of the pandemic. A second search was conducted to identify pre-existing influenza studies that were able to enrol A(H1N1)pdm09-infected patients. ClinicalTrials.gov was searched using the condition ‘influenza’. Detailed inclusion and exclusion criteria and subgroup analysis plans for both searches are contained in Additional file 1: appendix 3.

### Data extraction

One reviewer (AR) undertook data extraction according to the pre-specified inclusion and exclusion criteria. Decisions were recorded using electronic systematic review software (Rayyan [5]), available to the senior author (PWH). We did not request missing data from authors, as this does not contribute to the aims of this review. Details of the data extracted are in Additional file 1: appendix 3.

### Statistical analysis

Descriptive statistics are presented as frequencies for categorical variables and median with interquartile range for continuous variables. The findings from published literature and trial registries are reported separately. Analysis of the literature was stratified by research type. Chinese medicines are presented as a single class because individual components could not be differentiated. Assessment of combination therapy was not possible due to variable reporting practices in the literature. Stata MP/15.0 and Microsoft Excel for Mac/15.21.1 were used for statistical analysis and graphical depiction.

### Role of the funding source

The funder of the study had no role in study design, data collection, data analysis, data interpretation or writing of the report.

## Results

### Findings from published literature

**Fig. 1:**
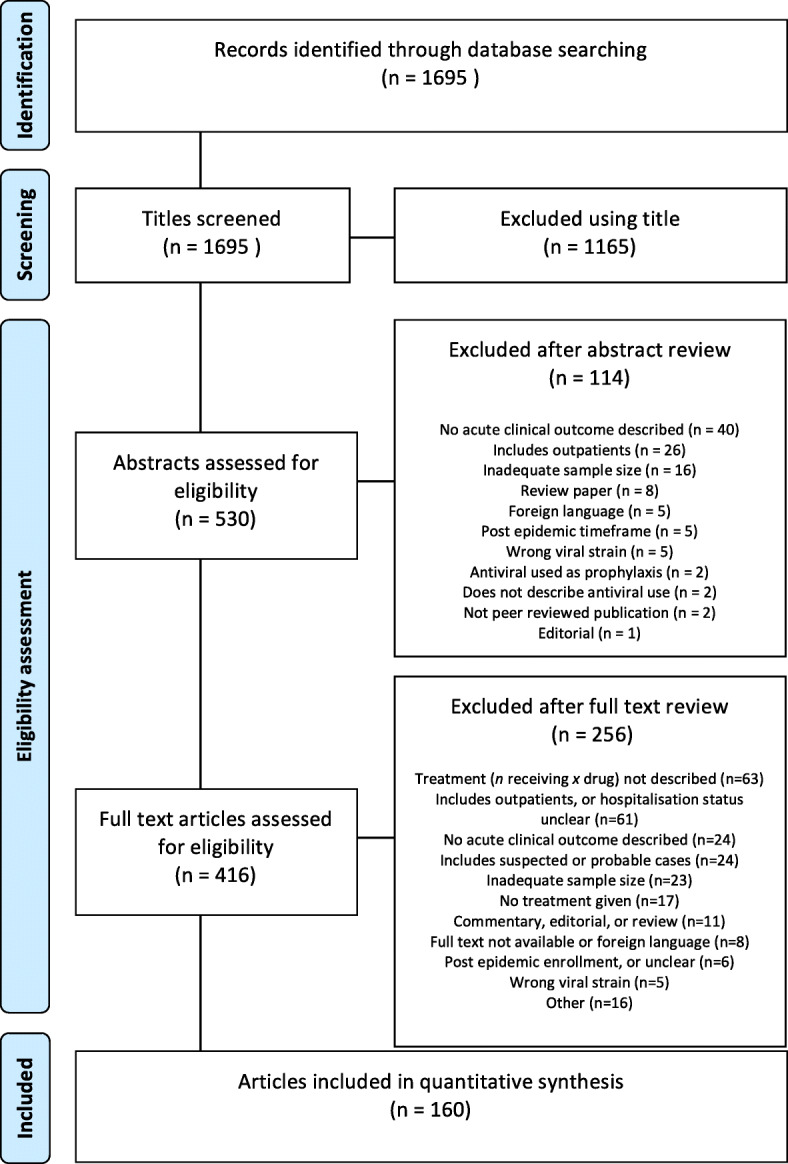
Study selection

This review includes 160 papers (summarised in Fig. 1, details in Additional file 1: appendix 4) that describe 39,577 hospitalised patients with A(H1N1)pdm09 and 33,869 treatment courses (Table 2). Twelve different treatments were used, with oseltamivir being the most common (Table 2). The median number of treatments described per manuscript is 63 (interquartile range, IQR 22–193). Of the 160 papers included, two are interventional trials (*n* = 73, representing 0.2% of total reported patients), [6, 7] 28 are prospective observational studies (*n* = 6102, accounting for 15.4% of total patients), 129 are retrospective observational studies or case reports (*n* = 33,342, 84.2% of total patients) and one enrolled patients both prospectively and retrospectively (*n* = 98, 0.2% of patients).

**Table 2:** Volume of treatment courses described in the literature for hospitalised patients

Treatment name	Number of publications reporting use	Total number of patients receiving treatment^**1**^	Median (IQR) number of patients receiving treatment per publication^**2**^	FDA drug approval status for use in influenza in 2009
Oseltamivir	154	31,737	63 (21–188)	Approved for acute uncomplicated influenza, expanded under EUA April 2009
Zanamivir	54	368	2 (1–9)	Approved for acute uncomplicated influenza, expanded under EUA April 2009
Peramivir	14	403	1 (1–3)	Unapproved, eIND in April 2009, EUA October 2009
Amantadine	11	86	3 (1–13)	Approved for acute uncomplicated influenza, but resistance to A(H1N1)pdm09 demonstrated
Rimantadine	5	32	3 (1–13)	Approved for acute uncomplicated influenza, but resistance to A(H1N1)pdm09 demonstrated
Ribavirin	5	34	2 (1–6)	Not approved for influenza
Intravenous immunoglobulin	4	44	4 (2–20)	Not approved for influenza
Chinese medicines	3	1051	245 (56–750)	Not approved for influenza
Convalescent Plasma	2	52	26	Not approved for influenza
Macrolides^3^	1	31	31	Not approved for influenza
Sirolimus	1	19	19	Not approved for influenza
Statins^3^	1	12	12	Not approved for influenza

Volume of treatment courses described in the literature for hospitalised patients with 2009 H1N1 influenza during the pandemic period

*eIND* emergency investigational new drug authorisation, *EUA* emergency use authorization

^1^Some patients received more than one treatment

^2^When publication describes use of that drug

^3^Where clear indication was influenza

Early initiation of prospective research maximises the probability of meeting sample size targets before an outbreak wanes. The median delay to first patient enrolment since the identification of the pandemic viral strain (April 1, 2009) for prospective observational studies was 102 days (IQR 61–172 days). The two clinical trials began enrolment after a delay of 244 and 275 days.

For prospective observational studies, enrolment stopped a median of 274 (IQR 195–313) days after viral identification. This was 223 days before the PHEIC ended (August 10, 2010), but when case numbers were falling. The two clinical trials closed enrolment 699 and 944 days after virus identification (March and November 2011).

**Fig. 2:**
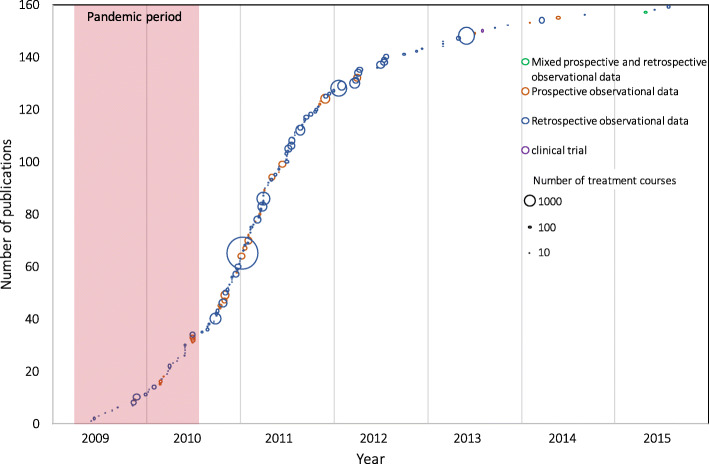
Publication of included studies over time. Studies are shown according to the type of the study and the number of treatment courses described. The pandemic period ranges from the 1st of April 2009 to the end of the PHEIC on 10th of August 2009

The publication dates of all articles over time are shown in Fig. 2. No (0/2) interventional trials were published before the end of the PHEIC. Twenty-five per cent (7/28) of prospective observational studies and 22% (28/130) of retrospective or mixed-enrolment research were published by the end of the PHEIC. The median date of publication for all papers was March 18, 2011 (IQR September 28, 2010, to October 24, 2011); this was 213 days after the PHEIC ended. Overall, the median delay between final patient enrolment (or inclusion) and date of publication was 444 days (IQR 281–684). The median date between final patient enrolment and submission was 302 days (IQR 142–534), between submission and acceptance 93 days (IQR 63–144), and acceptance to publication was 56 days (IQR 24–94) where data existed for these intervals.

Thirty-nine countries reported treatment data. The highest number of papers was published by the USA (*n* = 25, reporting 2559 treatment courses), followed by China (*n* = 16, reporting 14,680 treatment courses) and Spain (*n* = 16, reporting 4103 treatment courses). Country-level data describing the number of publications and treatment courses described and the first date of patient enrolment in prospective research (where relevant) are shown in Additional file 1: appendix 5).

Articles described the pregnancy status of patients in 88% (140/160) of articles. Articles described the inclusion of elderly patients in 88% (140/160) of articles and children in 93% (149/160) of articles.

Twenty-three per cent (36/160) of papers described adverse effects from treatment had occurred. In 42% of cases, adverse effects or severe adverse events were noted. Thirteen per cent (21/159) of articles tested for resistance and described some resistant samples, 4% (6/159) of articles tested for resistance but found no mutations, 3% (5/159) of articles reported clinical suspicion of antiviral resistance and, in the remaining 81% (129/159) of papers, there was no statement regarding antiviral resistance; one paper described no antiviral use and was excluded.

### Findings from H1N1 trial registrations

Fifteen H1N1 study registration records were included in the review (Additional file 1: appendix 6) comprising 10 interventional trials and 5 observational studies (2 with treatment efficacy outcomes, and 3 with general acute clinical outcomes) planned during the pandemic. A total of eight different treatments were to be studied: oseltamivir, zanamivir, convalescent plasma, intravenous immunoglobulin, rosuvastatin, sirolimus, Chinese herbs and vitamin supplementation (vitamins A, C and E).

**Fig. 3:**
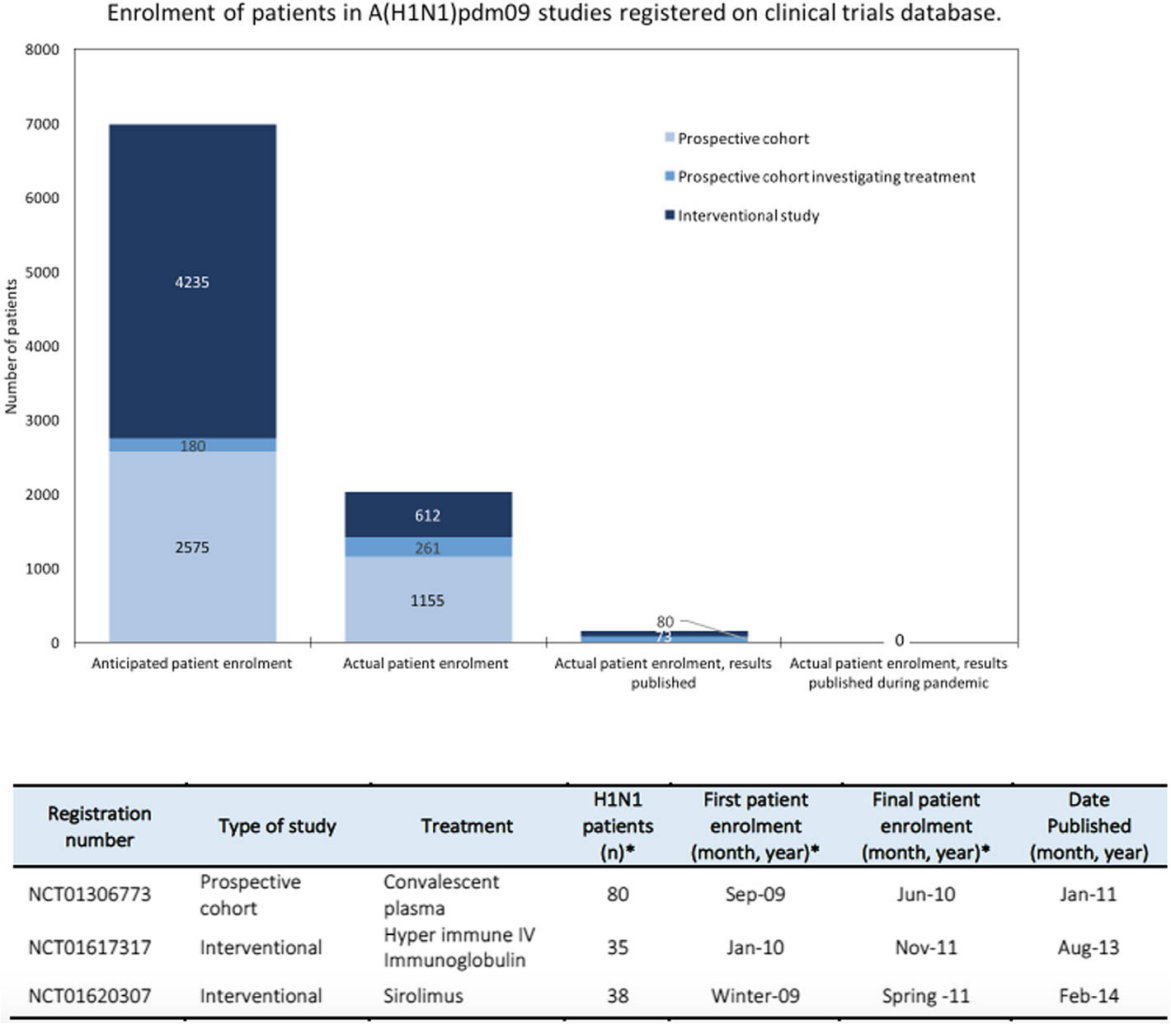
Anticipated and actual enrolment of patients in A(H1N1)pdm09 studies registered on clinical trials database. Table insert displays the enrolment number and publishing timeline for completed studies with results published. An asterisk denotes where conflict existed between numbers in the clinical trial record and publication, publication numbers were used

Of the 15 studies, nine are reported as completed, four were terminated due to the end of the H1N1 pandemic or declining case numbers and the status of two studies is not recorded. The anticipated and actual enrolment of patients into all studies is depicted in Fig. 3. Some study protocols excluded patients because of young age (25%) and pregnancy (50%). Results are available in the literature for three of the completed studies (Fig. 3), representing 153 patients, and available on the clinical trials registry for an additional two of the terminated studies. A sub-group analysis of clinical trials that only included hospitalised or severe cases is provided in Additional file 1: appendix 7.

### Findings from influenza trial registration

Eighteen influenza registration records were reviewed (Additional file 1: appendix 8). There were 16 interventional trials and two observational studies that were enrolling patients during the A(H1N1)pdm09 pandemic period. The treatments under investigation were oseltamivir, sambucol supplement, zanamivir, peramivir, amantadine, pomegranate supplement, nitazoxanide and favipiravir.

Eleven studies were completed, four were terminated early, the status of two is unknown and one study has ongoing enrolment listed. Results were available for nine of 11 completed studies (Table 3), representing 439 A(H1N1)pdm09 patients.

**Table 3:** Enrolment number and publishing timeline for completed studies

Registration number	Type of study	Treatment	Planned enrolment (***n***)	Total patients^**1**^ (***n***)	H1N1 patients^**1**^ (***n***)	First patient enrolment^**1**^ (month, year)	Last patient enrolment^**1**^ (month, year)	Date published (month, year)
**Enrolment commenced before pandemic**								
NCT00298233	Phase 2	Oseltamivir	400	326	72	Apr. 2007	Feb. 2010	May 2013 [8]
NCT00391768	Phase 1/2	Oseltamivir	108	87	37	Jan. 2007	Apr. 2010	Mar. 2013 [9]
**Enrolment commenced during pandemic**								
NCT00949533	Phase 3	Oseltamivir	125	37	Unknown	Aug. 2009	Oct. 2010	Apr. 2016 (Unp)
NCT00957996	Phase 3	Peramivir	300	127	94	Oct. 2009	Oct. 2010	Aug. 2013 [10]
NCT01199744	Prospective cohort	Zanamivir	N/R	1575	Unknown	Nov. 2009	Apr. 2010	Mar. 2011(Unp)
NCT01014988	Phase 2	Zanamivir	150	130	92	Nov. 2009	Sep. 2011	Feb. 2014 [11]
NCT01052961	Phase 4	Oseltamivir	400	155	34	Jan. 2010	Jun. 2012	Dec. 2013 [12]
NCT01050257	Phase 3	Oseltamivir	200	118	Unknown	Jan. 2010	Sep. 2012	Aug. 2013 (Unp)
NCT01068912	Phase 2	Favipiravir	384	530	110	Feb. 2010	May 2012	Feb. 2014 (Unp)

Enrolment number and publishing timeline for completed studies where results are published in the literature (date followed by reference) or on the clinical trials registration site (date followed by Unp)

*N/R* not reported

^1^Where conflict existed between numbers in the clinical trial record and publication, publication numbers were used

## Discussion

There is consistent criticism that the research response to disease outbreaks is fractured and delayed [13–15]. There has, however, been little quantitative examination of these assumed insufficiencies. This paper demonstrates that despite over 33,000 treatment courses being described for hospitalised patients with influenza A(H1N1)pdm09 during the pandemic, fewer than 600 received treatment (or placebo) as participants in a registered interventional clinical trial with results available in the peer-reviewed literature. None of these registered trial results was available during the timeframe of the pandemic, as were few of the findings from observational studies. This constitutes a significant failure to collect high-quality data.

Our findings demonstrate that we must make improvements in order to offer patients, and their treating clinicians, evidence-based care during pandemics, including the COVID-19 pandemic. Several drugs are being investigated as potential treatment for COVID-19, but we note that some early published studies were poorly controlled [16, 17]. It is imperative that we learn from the A(H1N1)pdm09 pandemic and ensure that trials of therapeutics are done under conditions which allow for the collection of high-quality, interpretable data to inform future clinical care.

We found that most descriptions of treatment courses were in retrospective observational studies or case series, with few prospective studies launched. There was relative success, however, in enrolling A(H1N1)pdm09 patients in ongoing or seasonal influenza studies—of the 582 patients enrolled in a trial, 439 were enrolled in this manner. Indeed, conducting trials of therapeutics for similar diseases (such as seasonal influenza) during an inter-epidemic period has been proposed as a solution to improving the outbreak research response [18]. This occurs not only by allowing the rapid recruitment of individuals in the case of an outbreak (as was observed here) but also by testing trial design and establishing research capacity. Novel trial designs, such as adaptive, platform trials, should also be adopted as a way to expedite outbreak research [18]. Using this approach, multiple treatments (or even multiple respiratory viruses) can be evaluated under an overarching protocol and regulatory framework, improving efficiency [19]. Findings presented here from 2009 support the need for this approach to respiratory virus pandemics, including COVID-19. Indeed, two large platform trials for therapeutics in COVID-19 are underway (RECOVERY [ISRCTN50189673] and Solidarity [ISRCTN83971151]) and already generating high-quality evidence [20]. Sleeper protocol research may provide a further solution. These pre-prepared and pre-approved protocols can lay dormant, waiting for cases of pandemic respiratory viruses, and allow prior assessment of the logistics and feasibility of the protocol. An example of this type of protocol exists for severe acute respiratory infections (NCT02498587) and has been used to rapidly enrol patients with COVID-19.

Recommendations following the Ebola virus disease (EVD) epidemic in West Africa suggest that clinical research should be initiated, enacted and completed by the time an epidemic peaks [21]. We found that from the time influenza A(H1N1)pdm09 was first detected, it was over 3 months before prospective data collection began and 8 months before the first interventional trial began recruitment. While these delays compare well to recent evaluations of delays for other epidemic observational research [22] or clinical trials [23], it remains too slow.

Additionally, the small sample sizes of literature included in our review indicate a fractured research response. It has been estimated that a sample size of 800 patients is required to power a randomised controlled trial of an NAI in hospitalised patients [24]. No prospective research identified here was that large. Beyond the benefits of increased enrolment and external validity, multicentre research has specific advantages in epidemics. It can compensate for unexpected variations in epidemiology at the regional level (such as the sudden end to the EVD outbreak in Liberia that prematurely halted a clinical treatment trial) [25] or the temporary closure of health care facilities with nosocomial transmission (such as occurred during the SARS outbreak of 2003) [26].

To ensure generalisability of findings, research responses to outbreaks should also be representative of geographic diversity and global epidemiology. In the results reported here, China was heavily represented and reported the majority of treatment courses described (14,680; 43.3% of the total). Patient characteristics (including age, gender and other risk factors) and healthcare systems vary substantially by country/region, with likely impacts on the suitability and relative efficacy of interventions in these different settings. A timely research response from one region will not necessarily be of benefit to the majority of the global at-risk population, particularly if low- and middle-income countries (LMICs) are poorly represented in the epidemic evidence base. Indeed, the importance of strengthening the research capacity and infrastructure of LMICs to enable effective outbreak responses was highlighted following the EVD epidemic [21, 27].

The inclusion of high-risk groups (such as elderly individuals, pregnant women and children) is also important to ensure generalisability of outbreak research. During the influenza A(H1N1)pdm09 pandemic, elderly and paediatric individuals were included in 88 and 93% of publications, respectively, noting again that the vast majority of these studies were observational only. At the onset of the pandemic, however, trials for approved neuraminidase inhibitors had only been conducted for mild seasonal influenza in healthy adults [28], without the inclusion of those highest at risk. A failure to include high-risk groups in initial prospective trials for novel medications may further delay the evidence base for these individuals when drugs are employed under outbreak circumstances.

We report long delays between clinical data capture and publication in the peer-reviewed literature. This is consistent with analyses for other disease outbreaks, including epidemiology reporting for SARS (where only 7% of articles were published within the timeframe of the epidemic) [29] and randomised controlled trials of pandemic H1N1 vaccines (where only 29% of clinical trial results were published almost a year following the end of the pandemic) [30]. The present WHO standard for interventional clinical trials is that main findings are to be submitted for publication within 12 months of study completion [31] and although no such guidelines exist for observational clinical data, there are analogous scientific and ethical imperatives for timely reporting.

While the ramifications of delayed reporting are described for other fields [32], there are specific imperatives for rapid data reporting during epidemics. For example, observational data must be accrued to design interventional trials (such as approximating the type and rate of outcomes). Emerging evidence can also prioritise trials so that the most promising continue recruitment when there are a declining number of cases late during an outbreak [18].

Initiatives to minimise publication delay now include fast-track review for manuscripts likely to change clinical practice [33]; this approach been widely employed by journals during the COVID-19 pandemic. Pre-approval of trial protocols (where some peer-review occurs before a study is conducted) or results-free review (where review excludes results and some discussion) are alternative peer-review models which aim to improve focus on study quality and reduce bias toward publication of only positive findings [34]. These may also have utility in speeding up the dissemination of quality clinical research in the outbreak setting, by allowing for peer-review to begin at the same time as data collection; whether this is the case is yet to be tested. There is also support for pre-publication online release of preliminary findings. Indeed, the increasing utilisation of pre-publication servers (such as medRxiv) has been reflected in the COVID-19 outbreak. Dissemination of research findings via pre-publication (prior to peer-review) reduces delays but carries risks for validation of methodology, accuracy of data and interpretation of findings. The extraordinary number of COVID-19 articles being submitted to pre-publication servers [35] has led to several rapid, open, peer-review platforms for COVID-19 preprints being developed [36, 37]. These aim to improve quality control in the period between preprint dissemination and the formal peer-review/publication process; such ‘overlay’ review models are new and how these will impact on the quality of data generation and dissemination is not yet known.

This systematic review has several limitations. The scope of our review was narrowed due to the high volume of clinical literature discussing A(H1N1)pdm09. In particular, we focused only on hospitalised patients where most antivirals were used [38]. There is likely to be considerable geographic variation in what illness severity necessitates hospitalisation which may have limited the inclusion of studies from countries where care models differ (including LMICs). Furthermore, only English language manuscripts were included which may have led to similar bias in included study geography. We included several publication types, including case series or observational studies to provide an estimate of patients who may have been eligible for inclusion in a clinical trial (noting this estimate does not represent the true number of hospitalised patients who were treated). The precision of any estimate is affected by excluding papers where treatment was not clearly defined and when pandemic strain influenza was not laboratory confirmed. Our estimates of patients enrolled in clinical trials is almost certainly an underestimation—much of the momentum toward compulsory registration of clinical trials [39] occurred subsequent to the pandemic and trials may have been registered elsewhere and so it is likely that other trials were planned, initiated or even completed without public knowledge. We restricted our observational data collection to that captured before the end of the PHEIC, and while we recognise that research continued to occur during the second and third waves of the epidemic, differentiating this work from routine seasonal influenza research became difficult. Finally, there was only one reviewer for pragmatic reasons.

## Conclusions

Here, we demonstrate how tolerance of treatment under compassionate care circumstances during the influenza A(H1N1)pdm09 pandemic was not matched with a commitment to capture high-quality data on treatment use and therefore failed the standards expected of modern evidence-based medicine. Moreover, we show that the data that was collected on patients was incompletely reported and published after prolonged delay. We recommend early initiation of multicentre collaborative trials and pre-approved or sleeper protocols as potential solutions to improve accumulation of treatment data during a pandemic.

## Supplementary information

**Additional file 1: Appendix 1.** Checklist summarising compliance with MOOSE and PRISMA guidelines. **Appendix 2.** Details of search strategy. **Appendix 3.** Details of clinical registry search strategy and data extraction. **Appendix 4.** Studies included in systematic review. **Appendix 5.** Country level data demonstrating number of treatment courses described in the literature, and date of first enrolment in prospective research, where relevant. **Appendix 6.** Prisma flow diagram describing screening, eligibility and inclusion for clinical trial registration records for studies aimed at enrolling A(H1N1)pdm09 patients. **Appendix 7.** Enrolment metrics for studies enrolling A(H1N1)pdm09patients, when analysis is restricted to ‘serious’ and hospitalized patients. **Appendix 8.** Prisma flow diagram describing screening, eligibility and inclusion for clinical trial registration records for studies aimed at enrolling influenza patients, that were open during the pandemic period.

## Data Availability

Details of all studies included in this systematic review are available in Additional file 1: appendix 4. Extracted data are available from the lead author on reasonable request.

## Abbreviations

COVID-19: Coronavirus disease 2019
EVD: Ebola virus disease
IQR: Interquartile range
LMICs: Low- and middle-income countries
MERS: Middle East respiratory syndrome
NAI: Neuraminidase inhibitor
PHEIC: Public Health Emergency of International Concern
SARS: Severe acute respiratory virus
WHO: World Health Organization
