# Supplementary material for: Compassionate drug (mis)use during pandemics: lessons for COVID-19 from 2009
Source: BMC Med. 2020 Aug 21;18:265. doi: 10.1186/s12916-020-01732-5 (PMC7441224; doi:10.1186/s12916-020-01732-5)

**Compassionate drug (mis)use during pandemics: lessons for COVID-19 from 2009.**

## Supplementary materials

**Contents:**

Appendix 1: Checklist summarising compliance with MOOSE and PRISMA guidelines

Appendix 2 – Details of search strategy

Appendix 3 – Details of clinical registry search strategy and data extraction

Appendix 4: Studies included in systematic review

Appendix 5: Country level data demonstrating number of treatment courses described in the literature, and date of first enrolment in prospective research, where relevant

Appendix 6: Prisma flow diagram describing screening, eligibility and inclusion for clinical trial registration records for studies aimed at enrolling A(H1N1)pdm09 patients.

Appendix 7: Enrolment metrics for studies enrolling A(H1N1)pdm09patients, when analysis is restricted to 'serious' and hospitalized patients

Appendix 8: Prisma flow diagram describing screening, eligibility and inclusion for clinical trial registration records for studies aimed at enrolling influenza patients, that were open during the pandemic period

## Appendix 1 – Compliance to MOOSE and PRISMA guidelines for reporting of systematic reviews

| **MOOSE GUIDELINES** | |
|---|---|
| **Reporting background should include** | |
| Problem definition | Yes |
| Hypothesis statement | N/A |
| Description | Yes |
| Type of exposure or intervention used | Yes |
| Type of study designs used | Yes |
| Study population | Yes |
| **Reporting of search strategy should include** | |
| Qualifications of searches (e.g. librarians and investigators) | Yes |
| Search strategy, including time period included in the synthesis and keywords | Yes |
| Effort to include all available studies, including contact with authors | No, discussed |
| Databases and registries searched | Yes |
| Search software used, name and version, including special features | Yes |
| Use of hand searching (e.g. reference lists of obtained articles) | No |
| List of citations located and those excluded including justification | Available on request |
| Method of addressing articles published in languages other than English | Yes |
| Method of handling abstracts and unpublished studies | No |
| Description of any contact with authors | No, discussed |
| **Reporting methods should include** | |
| Description of relevance or appropriateness of studies assembled for assessing the hypothesis to be tested | Yes |
| Rationale for the selection and coding of data (eg, sound clinical principles or convenience) | Yes |
| Documentation of how data were classified and coded (eg, multiple raters, blinding, and interrater reliability) | Yes |
| Assessment of confounding (eg, comparability of cases and controls in studies where appropriate) | N/A |
| Assessment of study quality, including blinding of quality assessors; stratification or regression on possible predictors of study results | N/A |
| Assessment of heterogeneity | N/A |
| Description of statistical methods (eg, complete description of fixed or random effects models, justification of whether the chosen models account for predictors of study results, dose-response models, or cumulative meta-analysis) in sufficient detail to be replicated | Yes |
| Provision of appropriate tables and graphics | Yes |
| **Reporting of results should include** | |
| Graphic summarizing individual study estimates and overall estimate | Yes, as appropriate |

| | |
|---|---|
| Table giving descriptive information for each study included | Yes |
| Results of sensitivity testing (eg, subgroup analysis) | Yes |
| Indication of statistical uncertainty of findings | Yes |
| **Reporting of discussion should include** | |
| Quantitative assessment of bias (eg, publication bias) | Yes |
| Justification for exclusion (eg, exclusion of non–English-language citations) | Yes |
| Assessment of quality of included studies | N/A |
| **Reporting of conclusions should include** | |
| Consideration of alternative explanations for observed results | Yes |
| Generalization of the conclusions (ie, appropriate for the data presented and within the domain of the literature review) | Yes |
| Guidelines for future research | Yes |
| Disclosure of funding source | Yes |

N/A: This is a bibliometic review and is not undertaken to assess a treatment effect, and so this item is not applicable.

| PRISMA GUIDELINES | | | |
|---|---|---|---|
| **Section/topic** | **#** | **Checklist item** | **Reported on page #** |
| **Title** | | | |
| Title | 1 | Identify the report as a systematic review, meta-analysis, or both. | 1 |
| **Abstract** | | | |
| Structured summary | 2 | Provide a structured summary including, as applicable: background; objectives; data sources; study eligibility criteria, participants, and interventions; study appraisal and synthesis methods; results; limitations; conclusions and implications of key findings; systematic review registration number. | 1 |
| **Introduction** | | | |
| Rationale | 3 | Describe the rationale for the review in the context of what is already known. | 2 |
| Objectives | 4 | Provide an explicit statement of questions being addressed with reference to participants, interventions, comparisons, outcomes, and study design (PICOS). | N/A |
| **Methods** | | | |
| Protocol and registration | 5 | Indicate if a review protocol exists, if and where it can be accessed (e.g., Web address), and, if available, provide registration information including registration number. | 3 |
| Eligibility criteria | 6 | Specify study characteristics (e.g., PICOS, length of follow-up) and report characteristics (e.g., years considered, language, publication status) used as criteria for eligibility, giving rationale. | 3 |
| Information sources | 7 | Describe all information sources (e.g., databases with dates of coverage, contact with study authors to identify additional studies) in the search and date last searched. | 3 |
| Search | 8 | Present full electronic search strategy for at least one database, including any limits used, such that it could be repeated. | 3 |
| Study selection | 9 | State the process for selecting studies (i.e., screening, eligibility, included in systematic review, and, if applicable, included in the meta-analysis). | 3 |
| Data collection process | 10 | Describe method of data extraction from reports (e.g., piloted forms, independently, in duplicate) and any processes for obtaining and confirming data from investigators. | 3 |
| Data items | 11 | List and define all variables for which data were sought (e.g., PICOS, funding sources) and any assumptions and simplifications made. | 3 |
| Risk of bias in individual studies | 12 | Describe methods used for assessing risk of bias of individual studies (including specification of whether this was done at the study or outcome level), and how this information is to be used in any data synthesis. | N/A |
| Summary measures | 13 | State the principal summary measures (e.g., risk ratio, difference in means). | 4 |
| Synthesis of results | 14 | Describe the methods of handling data and combining results of studies, if done, including measures of consistency (e.g., $I^2$) for each meta-analysis. | N/A |
| Risk of bias across studies | 15 | Specify any assessment of risk of bias that may affect the cumulative evidence (e.g., publication bias, selective reporting within studies). | N/A |
| Additional analyses | 16 | Describe methods of additional analyses (e.g., sensitivity or subgroup analyses, meta-regression), if done, indicating which were pre-specified. | 3 |
| **Results** | | | |
| Study selection | 17 | Give numbers of studies screened, assessed for eligibility, and included in the review, with reasons for exclusions at each stage, ideally with a flow diagram. | Figure 2 |
| Study characteristics | 18 | For each study, present characteristics for which data were extracted (e.g., study size, PICOS, follow-up period) and provide the citations. | Appendix 4 |

| PRISMA GUIDELINES | | | |
|---|---|---|---|
| **Section/topic** | **#** | **Checklist item** | **Reported on page #** |
| Risk of bias within studies | 19 | Present data on risk of bias of each study and, if available, any outcome level assessment (see item 12). | N/A |
| Results of individual studies | 20 | For all outcomes considered (benefits or harms), present, for each study: (a) simple summary data for each intervention group (b) effect estimates and confidence intervals, ideally with a forest plot. | 5-6 as appropriate |
| Synthesis of results | 21 | Present results of each meta-analysis done, including confidence intervals and measures of consistency. | N/A |
| Risk of bias across studies | 22 | Present results of any assessment of risk of bias across studies (see Item 15). | N/A |
| Additional analysis | 23 | Give results of additional analyses, if done (e.g., sensitivity or subgroup analyses, meta-regression [see Item 16]). | Appendix 2 |
| **Discussion** | | | |
| Summary of evidence | 24 | Summarize the main findings including the strength of evidence for each main outcome; consider their relevance to key groups (e.g., healthcare providers, users, and policy makers). | 7-8 |
| Limitations | 25 | Discuss limitations at study and outcome level (e.g., risk of bias), and at review-level (e.g., incomplete retrieval of identified research, reporting bias). | 9 |
| Conclusions | 26 | Provide a general interpretation of the results in the context of other evidence, and implications for future research. | 9 |
| **Funding** | | | |
| Funding | 27 | Describe sources of funding for the systematic review and other support (e.g., supply of data); role of funders for the systematic review. | 10 |

N/A: This is a bibliometic review and is not undertaken to assess a treatment effect, and so this item is not applicable.

**Appendix 2 – Search Strategy**

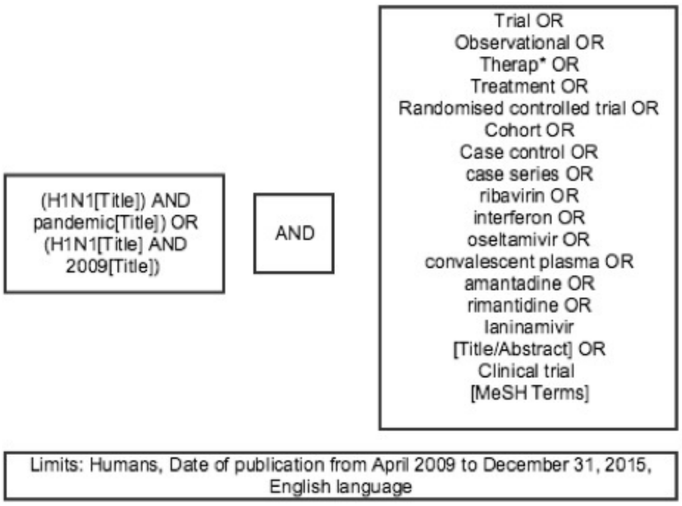

**Appendix 3: Details of clinical registry search strategy and data extraction**

*Search 1: Search of clinical trial records for A(H1N1)pdm09 influenza records.*

We searched clinicaltrials.gov using the term "H1N1". We included submissions submitted after the date of onset of the pandemic, and that began recruitment during the pandemic period, and were available on the database as of the 31st of December 2015. We included clinical trials and observational research enrolling patients with confirmed A(H1N1)pdm09. Trials must have reported on one or more outcomes of drug safety, efficacy, pharmacokinetics, or patient clinical outcomes. There was no minimum anticipated, or actual sample size. We excluded submissions where A(H1N1)pdm09 influenza was not specifically identified as a strain of interest. Registration records infrequently defined the hospitalisation status of patients, and so a subgroup analysis limiting enrolment to when records were explicit about enrolling only 'severe' or hospitalised patients is found in appendix 3 (supplementary figure 1).

*Search 2: Search of clinical trial records for influenza records*

We searched clinicaltrials.gov using the term "influenza" with Boolean operators to exclude "vaccine", "vaccination", "prophylaxis", and "diagnostic". We included submissions if they began recruitment before or during the pandemic (so long as they remained open to enrolment for some period during the pandemic period) and were available on the database as of the 31st of December 2015. We included clinical trials and observational research enrolling patients with confirmed influenza. Trials must have reported on one or more outcomes of drug safety, efficacy, pharmacokinetics, or patient clinical outcomes. There was no minimum anticipated, or actual sample size. Analysis is stratified as to whether the studies were ongoing at the time of the pandemic onset (April 1 2009).

*Details of data extraction*

For published literature, we extracted the type of research, the location of research, patient outcome measure(s) described, the number of patients, the dates that patients were enrolled, the number of patients receiving A(H1N1)pdm09 specific treatments, reporting of adverse events, reporting of drug resistance, the dates for manuscript submission, acceptance and first publication (including e-publication), and information on inclusion of some high-risk groups - pregnant women, the elderly (defined as older than 65 years), and children (defined as younger than 18 years). Where dates were only described to the closest month, the first day of the month was entered.

For registered clinical trials, we included the type of study, date of submission to the registry, predicted dates of enrolment and sample size (and then actual values when known), whether high-risk groups (as defined above) were included, the geographical location of the research, the completion status of the work, and the date of first publication (including e-publication). These details were cross checked against any corresponding publications.

## Appendix 4 – Details of literature included in this systematic review

| First author name | Study name | *n* |
|---|---|---|
| **Prospective research** | | |
| Al-Tawfiq | Pandemic influenza A (2009 H1N1) in hospitalized patients in a Saudi Arabian hospital: Epidemiology and clinical comparison with H1N1-negative patients | 47 |
| Beutel | Virus-associated hemophagocytic syndrome as a major contributor to death in patients with 2009 influenza A (H1N1) infection. | 25 |
| Champunot | Impact of Pandemic Influenza (H1N1) Virus Associated Community Acquired Pneumonia among adults in a tertiary hospital in Thailand | 24 |
| Chitnis | Epidemiologic and clinical features among patients hospitalized in Wisconsin with 2009 H1N1 influenza A virus infections, April to August 2009. | 252 |
| Cordero | Immunosuppressed patients with pandemic influenza A 2009 (H1N1) virus infection | 559 |
| Cordero | Pandemic influenza A(H1N1) virus infection in solid organ transplant recipients: impact of viral and non-viral co-infection | 51 |
| Denholm | Hospitalised adult patients with pandemic (H1N1) 2009 influenza in Melbourne, Australia | 112 |
| Diaz | Impact of Obesity in Patients Infected With 2009 Influenza A(H1N1) | 416 |
| Giannella | Prolonged viral shedding in pandemic influenza A(H1N1): clinical significance and viral load analysis in hospitalized patients | 64 |
| Hung | Convalescent Plasma Treatment Reduced Mortality in Patients With Severe Pandemic Influenza A (H1N1) 2009 Virus Infection | 93 |
| Hung, | Hyperimmune IV immunoglobulin treatment: a multicenter double-blind randomized controlled trial for patients with severe 2009 influenza A(H1N1) infection | 35 |
| Kirakli | Survival from severe pandemic H1N1 in urban and rural Turkey: a case series | 18 |
| Koegelenerg | High mortality from respiratory failure secondary to swine-origin influenza A (H1N1) in South Africa | 19 |
| Lee | Complications and Outcomes of Pandemic 2009 Influenza A (H1N1) Virus Infection in Hospitalized Adults: How Do They Differ From Those in Seasonal Influenza | 382 |
| Ling | Effects of Early Oseltamivir Therapy on Viral Shedding in 2009 Pandemic Influenza A (H1N1) Virus Infection | 70 |
| Lucker | Clinical features and outcomes of hospitalised adults and children with the 2009 influenza A H1N1 infection at Geneva's University Hospital | 85 |
| Martin - Loeches | Severe pandemic (H1N1)v influenza A infection: report on the first deaths in Spain | 131 |
| Martin - Loeches | Pandemic and post-pandemic Influenza A (H1N1) infection in critically ill patients | 648 |
| Ploin | Complications and factors associated with severity of influenza in hospitalized children and adults during the pandemic wave of A(H1N1)pdm2009 infections--the Fluco French cohort | 40 |
| Poeppl | Clinical aspects of 2009 pandemic influenza A (H1N1) virus infection in Austria | 450 |
| Riquelme | Characteristics of hospitalised patients with 2009 H1N1 influenza in Chile | 100 |
| Rodriguez | Impact of early oseltamivir treatment on outcome in critically ill patients with 2009 pandemic influenza | 657 |
| Stein | Hospitalization of Children With Influenza A(H1N1) Virus in Israel During the 2009 Outbreak in Israel | 478 |
| Suarez-Varela | Pandemic influenza A (H1N1) infection in pregnant and nonpregnant women in Spain (2009-2010). | 219 |
| Viasus | Factors associated with severe disease in hospitalized adults with pandemic (H1N1) 2009 in Spain | 585 |

| First author name | Study name | *n* |
|---|---|---|
| Viasus, Pano-Pardo | Effect of immunomodulatory therapies in patients with pandemic influenza A (H1N1) 2009 complicated by pneumonia | 197 |
| Wang | Adjuvant Treatment with a Mammalian Target of Rapamycin Inhibitor, Sirolimus, and Steroids Improves Outcomes in Patients with Severe H1N1 Pneumonia and Acute Respiratory Failure | 38 |
| Wu | Severity of pandemic H1N1 2009 influenza virus infection may not be directly correlated with initial viral load in upper respiratory tract | 18 |
| Yates | Influenza A/H1N1v in pregnancy: an investigation of the characteristics and management of affected women and the relationship to pregnancy outcomes for mother and infant | 241 |
| Yung | Pandemic H1N1 in children requiring intensive care in Australia and New Zealand during winter 2009 | 83 |
| **Retrospective research** | | |
| Al-Lawati | Hospitalised patients with Influenza A (H1N1) in the Royal Hospital, Oman: Experience of a tertiary care hospital, July-December 2009 | 131 |
| Altmann | Severe Cases of Pandemic (H1N1) 2009 in Children, Germany | 93 |
| Asselet | Clinical, virological and epidemiological assessment of 2009 influenza A (H1N1) pandemic in a Belgian university hospital | 28 |
| Bagdure | Hospitalized Children with 2009 Pandemic Influenza A (H1N1): Comparison to Seasonal Influenza and Risk Factors for Admission to the ICU | 307 |
| Balanzat | An analysis of 332 fatalities infected with pandemic 2009 influenza A (H1N1) in Argentina | 332 |
| Baltagi | Neurological sequelae of 2009 influenza A (H1N1) in children: A case series observed during a pandemic | 1 |
| Brandsaeter | Hospitalised patients with suspected 2009 H1N1 influenza A in a hospital in Norway, July - December 2009 | 64 |
| Calitri | Clinical features of hospitalised children with 2009 H1N1 influenza virus infection | 63 |
| Camous | Clinical features of H1N1 2009 infection in critically ill immunocompromised patients | 10 |
| Campbell | Respiratory Failure Caused by 2009 Novel Influenza A/H1N1 in a Hematopoietic Stem-Cell Transplant Recipient: Detection of Extrapulmonary H1N1 RNA and Use of Intravenous Peramivir | 1 |
| Cao | Clinical Features of the Initial Cases of 2009 Pandemic Influenza A (H1N1) Virus Infection in China | 426 |
| CDC | Neurologic complications associated with novel influenza A (H1N1) virus infection in children - Dallas, Texas, May 2009 | 4 |
| Chacko | Cardiac manifestations in patients with pandemic (H1N1) 2009 virus infection needing intensive care | 37 |
| Chan | Pandemic (H1N1) 2009: clinical and laboratory findings of the first fifty cases in Singapore | 50 |
| Chan | Distinguishing characteristics between pandemic 2009-2010 influenza A (H1N1) and other viruses in patients hospitalized with respiratory illness. | 291 |
| Cheng | Clinical and epidemiological profile of patients with severe H1N1/09 pandemic influenza in Australia and New Zealand: an observational cohort study | 478 |
| Chien | Predictors and outcomes of respiratory failure among hospitalized pneumonia patients with 2009 H1N1 influenza in Taiwan | 96 |
| Chiu | Age-matched comparison of children hospitalized for 2009 pandemic H1N1 influenza with those hospitalized for seasonal H1N1 and H3N2 | 99 |
| Choi | Critically Ill Patients with Pandemic Influenza A/H1N1 2009 at a Medical Center in Korea | 27 |
| Chong | Risk Factors for Complicated Influenza A (H1N1) 2009 Disease in Children | 143 |

| First author name | Study name | *n* |
|---|---|---|
| Chowell | Risk factors for mortality among 2009 A/H1N1 influenza hospitalizations in Maricopa County, Arizona, April 2009 to March 2010 | 546 |
| Chudasama | Hospitalizations associated with 2009 influenza A (H1N1) and seasonal influenza in Saurashtra region, India | 274 |
| Chudasama | Characteristics of Fatal Cases of Pandemic Influenza A (H1N1) from September 2009 to January 2010 in Saurashtra Region, India | 71 |
| Chudasama | Correlates of severe disease in patients admitted with 2009 pandemic influenza A (H1N1) infection in Saurashtra region, India | 274 |
| Clay | Postpartum pharmacokinetics of peramivir in the treatment of 2009 H1N1 influenza | 1 |
| Creanga | Severity of 2009 Pandemic Influenza A (H1N1) Virus Infection in Pregnant Women | 136 |
| Cui | Factors associated with death in hospitalized pneumonia patients with 2009 H1N1 influenza in Shenyang, China | 68 |
| Da Dalt | Oseltamivir-resistant pandemic (H1N1) 2009 treated with nebulized zanamivir | 1 |
| Da Dalt | Pandemic influenza A (H1N1v) infection in pediatric population: a multicenter study in a north-east area of Italy | 200 |
| del Rosal | Pandemic H1N1 influenza-associated hospitalizations in children in Madrid, Spain | 517 |
| Delgado-Rodriguez | Prognosis of hospitalized patients with 2009 H1N1 influenza in Spain: influence of neuraminidase inhibitors | 813 |
| Dubar | French Experience of 2009 A/H1N1v Influenza in Pregnant Women | 151 |
| Dulek | Use of intravenous zanamivir after development of oseltamivir resistance in a critically Ill immunosuppressed child infected with 2009 pandemic influenza A (H1N1) virus | 1 |
| Englund | Oseltamivir-resistant novel influenza A (H1N1) virus infection in two immunosuppressed patients - Seattle, Washington, 2009 | 2 |
| Enstone | Nosocomial Pandemic (H1N1) 2009, United Kingdom, 2009–2010 | 30 |
| Ergin | Clinical and epidemiological features of Turkish children with 2009 pandemic influenza A (H1N1) infection: Experience from multiple tertiary paediatric centres in Turkey | 821 |
| Esposito | Oseltamivir-induced resistant pandemic A/H1N1 influenza virus in a child with cystic fibrosis and Pseudomonas aeruginosa infection | 18 |
| Fraaij | Evaluation of the Antiviral Response to Zanamivir Administered Intravenously for Treatment of Critically Ill Patients With Pandemic Influenza A (H1N1) Infection | 13 |
| Fuhrman | Adult intensive-care patients with 2009 pandemic influenza A(H1N1) infection | 1065 |
| Fujikura | The (H1N1) 2009 Pandemic Influenza Pneumonia among Adult Patients in Japan | 346 |
| Gaur | Intravenous zanamivir for oseltamivir-resistant 2009 H1N1 influenza | 1 |
| Ghosh | Efficient control of pandemic 2009 H1N1 virus infection with intravenous zanamivir despite the lack of immune function | 1 |
| Gonzalez-Velez | Factors associated to admission to Intensive Care in patients hospitalized due to pandemic Influenza A/H1N1 2009 | 100 |
| Hagerman | Clinical characteristics and outcomes in children hospitalised with pandemic influenza A/H1N1/09 virus infection – a nationwide survey by the Pediatric Infectious Diseases Group of Switzerland | 326 |
| Harter | Intravenous zanamivir for patients with pneumonitis due to pandemic (H1N1) 2009 influenza virus | 226 |

| First author name | Study name | *n* |
|---|---|---|
| Hasegawa | Characteristics of atopic children with pandemic H1N1 influenza viral infection: Pandemic H1N1 influenza reveals 'occult' asthma of childhood | 33 |
| Hasegawa | Pandemic (H1N1) 2009–associated Pneumonia in Children, Japan | 80 |
| Heninger | Comparative Analysis of Clinical Characteristics of Pandemic Influenza A/H1n1 and Seasonal Influenza A Infections in Hospitalized Children | 57 |
| Hernandez | Clinical Experience in Adults and Children Treated with Intravenous Peramivir for 2009 Influenza A (H1N1) Under an Emergency IND Program in the United States | 42 |
| Hiba | Benefit of early treatment with oseltamivir in hospitalized patients with documented 2009 influenza A (H1N1): retrospective cohort study | 502 |
| Hong-Ryang Kil | Early corticosteroid treatment for severe pneumonia caused by 2009 H1N1 influenza virus | 32 |
| Iglesias | Reducing occurrence and severity of pneumonia due to pandemic H1N1 2009 by early oseltamivir administration: a retrospective study in Mexico | 442 |
| Ismail | Characteristics of Children Hospitalized for Pandemic (H1N1) 2009, Malaysia | 1321 |
| Jain | Hospitalized Patients with 2009 H1N1 Influenza in the United States, April–June 2009 | 272 |
| Kang | Virological Clearance Rate of High-Dose Oseltamivir or Triple-Combination Antiviral Therapy in Complicated 2009 Pandemic Influenza A (H1N1) Infection | 14 |
| Kar-Hui | Benign Clinical Course in H1N1 2009 Influenza Infection in Young Oseltamivir-treated Immunocompromised Patients With Kidney Disease in Singapore | 38 |
| Kedia | Pediatric neurological complications of 2009 pandemic influenza A (H1N1) | 23 |
| Kendirli | Critically ill children with pandemic influenza (H1N1) in pediatric intensive care units in Turkey | 83 |
| Khandaker | Nosocomial vs community-acquired pandemic influenza A (H1N1) 2009: a nested case-control study | 188 |
| Kim | Triple-combination antiviral drug for pandemic H1N1 influenza virus infection in critically ill patients on mechanical ventilation. | 127 |
| Kim | Fatal cases of 2009 pandemic influenza A (H1N1) in Korea | 115 |
| Kinikar | Predictors of mortality in hospitalized children with pandemic H1N1 influenza 2009 in Pune, India | 92 |
| Ko | Characteristics of hospitalized children with 2009 pandemic influenza A (H1N1): a multicenter study in Korea | 804 |
| Kusznierz | Clinical features of the hospitalized patients with 2009 pandemic influenza A (H1N1) in Santa Fe, Argentina | 242 |
| Kute | High mortality in critically ill patients infected with 2009 pandemic influenza A (H1N1) with pneumonia and acute kidney injury | 20 |
| Lam | Severe cases of pandemic H1N1 pneumonia and respiratory failure requiring intensive care | 18 |
| Larcombe | Pandemic (H1N1) 2009: a clinical spectrum in the general paediatric population | 43 |
| Launes | 2009 Influenza A H1N1 infections: delays in starting treatment with oseltamivir were associated with a more severe disease | 127 |
| Law | Severe refractory hypoxaemia in H1N1 (2009) intensive care patients: initial experience in an Asian regional hospital | 12 |
| Lee | Fatalities associated with the 2009 H1N1 influenza A virus in New York city | 123 |
| Li | Clinical features of 2009 pandemic influenza A (H1N1) virus infection in chronic hemodialysis patients | 15 |

| First author name | Study name | *n* |
|---|---|---|
| Liang | New influenza A (H1N1) 2009 in Singapore: the first ten adult imported cases | 10 |
| Liu | Sixty-two severe and critical patients with 2009 influenza A (H1N1) in Shanghai, China | 62 |
| Low | Epidemiological and Clinical Features of 308 Hospitalized Patients with Novel 2009 Influenza A (H1N1) Virus Infection in China during the First Pandemic Wave | 22 |
| Mady | Clinical experience with severe 2009 H1N1 influenza in the intensive care unit at King Saud Medical City, Saudi Arabia | 86 |
| Memoli | Rapid selection of oseltamivir- and peramivir-resistant pandemic H1N1 virus during therapy in 2 immunocompromised hosts | 2 |
| Mickiene | Hospitalized adult patients with 2009 pandemic influenza A (H1N1) in Kaunas, Lithuania | 121 |
| Miller | Clinical Findings and Demographic Factors Associated With ICU Admission in Utah Due to Novel 2009 Influenza A(H1N1) Infection | 47 |
| Minnema | Comparison of Hospitalized Solid Organ Transplant Recipients and Nonimmunocompromised Patients With Pandemic H1N1 Infection: A Retrospective Cohort Study | 17 |
| Minnema | Clinical characteristics and outcome associated with pandemic (2009) H1N1 influenza infection in patients with hematologic malignancies: a retrospective cohort study | 64 |
| Moore | Evidence of Person-to-Person Transmission of Oseltamivir-Resistant Pandemic Influenza A(H1N1) 2009 Virus in a Hematology Unit | 10 |
| Moral | Burden of Severe 2009 Pandemic Influenza A (H1N1) Infection in Children in Southeast Spain | 82 |
| Nassar | Influenza A (H1N1) patients admitted to intensive care units during the 2009 pandemics: clinical features and outcomes | 22 |
| Nermin | A retrospective evaluation of critically ill patients infected with H1N1 influenza A virus in Bursa, Turkey, during the 2009–2010 pandemic | 23 |
| No authors | Human infection with new influenza A (H1N1) virus: clinical observations from a school-associated outbreak in Kobe, Japan, May 2009 | 49 |
| Noun | Pandemic (H1N1) 2009 influenza in pediatric hematology/oncology units in Lebanon | 14 |
| O'Riordan | Risk factors and outcomes among children admitted to hospital with pandemic H1N1 influenza | 58 |
| Oh | A Prediction Rule to Identify Severe Cases among Adult Patients Hospitalized with Pandemic Influenza A (H1N1) 2009 | 709 |
| Okada | Characteristic findings of pediatric inpatients with pandemic (H1N1) 2009 virus infection among severe and nonsevere illnesses | 46 |
| Owayed | Epidemiology and clinical presentation of pandemic influenza A (H1N1) among hospitalized children in Kuwait | 197 |
| Parakh | Pediatric hospitalizations associated with 2009 pandemic influenza A (H1N1): an experience from a tertiary care center in north India | 25 |
| Park | Successful salvage therapy with inhaled zanamivir in a patient with peramivir-resistant pandemic influenza A (H1N1) 2009 virus | 1 |
| Perez - Padilla | Pneumonia and respiratory failure from swine-origin influenza A (H1N1) in Mexico | 18 |
| Petersen | Failure of combination oral oseltamivir and inhaled zanamivir antiviral treatment in ventilator- and ECMO-treated critically ill patients with pandemic influenza A (H1N1)v | 21 |
| Peterson | Failure of combination oral oseltamivir and inhaled zanamivir antiviral treatment in ventilator- and ECMO-treated critically ill patients with pandemic influenza A (H1N1) | 21 |

| **First author name** | **Study name** | ***n*** |
|---|---|---|
| Regan | Epidemiology of influenza A (H1N1)pdm09-associated deaths in the United States, September–October 2009 | 323 |
| Rello | Intensive care adult patients with severe respiratory failure caused by Influenza A (H1N1)v in Spain | 32 |
| Rhim | Pandemic 2009 H1N1 virus infection in children and adults: A cohort study at a single hospital throughout the epidemic | 373 |
| Rhim | Epidemiological and clinical characteristics of childhood pandemic 2009 H1N1 virus infection: An observational cohort study | 217 |
| Rojas Surarez | Maternal mortality due to pandemic influenza A H1N1 2009 virus in Colombia | 23 |
| Schellongowski | A surge of flu-associated adult respiratory distress syndrome in an Austrian tertiary care hospital during the 2009/2010 Influenza A H1N1v pandemic | 17 |
| Sertogullarindan | Clinical and prognostic features of patients with pandemic 2009 influenza A (H1N1) virus in the intensive care unit | 20 |
| Shetty | Oseltamivir-resistant 2009 H1N1 influenza pneumonia during therapy in a renal transplant recipient | 1 |
| Siau | Influenza A H1N1 (2009): clinical spectrum of disease among adult patients admitted to a regional hospital in Singapore | 153 |
| Siau | Severe refractory hypoxaemia in H1N1 (2009) intensive care patients: initial experience in an Asian regional hospital | 12 |
| Soonhak | Neurologic Complications and Outcomes of Pandemic (H1N1) 2009 in Korean Children | 23 |
| Sorbello | Emergency use authorization for intravenous peramivir: evaluation of safety in the treatment of hospitalized patients infected with 2009 H1N1 influenza A virus | 344 |
| Souza | H1N1pdm Influenza Infection in Hospitalized Cancer Patients: Clinical Evolution and Viral Analysis | 24 |
| Sugaya | Efficacy, Safety, and Pharmacokinetics of Intravenous Peramivir in Children with 2009 Pandemic H1N1 Influenza A Virus Infection | 106 |
| Suhalia | Mixtures of oseltamivir-sensitive and -resistant pandemic influenza A/H1N1/2009 viruses in immunocompromised hospitalized children | 1 |
| Surana | Neurological complications of pandemic influenza A H1N1 2009 infection: European case series and review | 1 |
| Tabarsi | Factors associated with death or intensive care unit admission due to pandemic 2009 influenza A (H1N1) infection | 46 |
| To | Concurrent comparison of epidemiology, clinical presentation and outcome between adult patients suffering from the pandemic influenza A (H1N1) 2009 virus and the seasonal influenza A virus infection | 69 |
| van Zwol | Clinical Features of a Dutch Cohort of Critically Ill Children Due to the 2009 New Influenza A H1N1 Pandemic | 13 |
| Venkata | Hospitalized patients with 2009 H1N1 influenza infection: the Mayo Clinic experience | 66 |
| Webster | Oseltamivir-resistant pandemic H1N1 influenza | 1 |
| Wieching | Clinical characteristics of pediatric hospitalizations associated with 2009 pandemic influenza A (H1N1) in Northern Bavaria, Germany | 94 |
| Wijaya | Intravenous zanamivir in critically ill patients due to pandemic 2009 (H1N1) influenza A virus | 5 |
| Wolfe | Pandemic (H1N1) 2009 and Oseltamivir Resistance in Hematology/Oncology Patients | 1 |
| Xi | Hospitalized adult patients with 2009 influenza A(H1N1) in Beijing, China: risk factors for hospital mortality | 155 |

| **First author name** | **Study name** | ***n*** |
|---|---|---|
| Xiao | Hospitalized patients with novel influenza A (H1N1) virus infection: Shanghai, June–July 2009 | 156 |
| Yang | Antiviral therapy and outcomes of patients with pneumonia caused by influenza a pandemic (H1N1) virus. | 1461 |
| Yu | Risk factors for severe illness with 2009 pandemic influenza A (H1N1) virus infection in China | 9966 |
| Yu | Effectiveness of oseltamivir on disease progression and viral RNA shedding in patients with mild pandemic 2009 influenza A H1N1: opportunistic retrospective study of medical charts in China | 1291 |
| Zhang | Risk factors for adult death due to 2009 pandemic influenza A (H1N1) virus infection: a 2151 severe and critical cases analysis | 2151 |
| Zhang | Clinical features and risk factors for severe and critical pregnant woment with 2009 pandemic H1N1 influenza infection in China | 394 |
| Zhang | Epidemiological and Clinical Features of 308 Hospitalized Patients with Novel 2009 Influenza A (H1N1) Virus Infection in China during the First Pandemic Wave | 308 |
| Zheng | Hospitalized children with 2009 influenza A (H1N1) infection in Shenzhen, China, November-December 2009 | 148 |
| Zhou | Clinical features of initial cases of 2009 pandemic influenza A (H1N1) in Macau, China. | 72 |
| **Mixed prospective and retrospective research** | | |
| Prerna | Neurology of the H1N1 pandemic in Singapore: a nationwide case series of children and adults | 98 |

Appendix 4: Studies included in systematic review. n is the number of patients with laboratory confirmed influenza A(H1N1) pdm09 included in analysis. This excludes oseltamivir use when fewer than ten patients were treated.

| Country reporting data | Number of treatment courses described in literature (by study enrolment type) | | | Total number of papers published | Date of first patient enrolment in prospective research |
|---|---|---|---|---|---|
| | Prospective | Retrospective | Total | | |
| Argentina | | 517 | 517 | 2 | |
| Australia | 95 | 126 | 221 | 3 | 01/05/2009 |
| Australia and New Zealand | 67 | 390 | 457 | 2 | 01/06/2009 |
| Austria | 355 | 9 | 364 | 2 | 20/09/2009 |
| Belgium | | 27 | 27 | 1 | |
| Brazil | | 17 | 17 | 1 | |
| Canada | | 98 | 98 | 4 | |
| Chile | 97 | | 97 | 1 | 29/05/2009 |
| China | 384 | 14296 | 14680 | 16 | 01/06/2009 |
| Colombia | | 26 | 26 | 1 | |
| Denmark | | 84 | 84 | 2 | |
| France | 38 | 770 | 808 | 4 | 30/11/2009 |
| Germany | 39 | 79 | 118 | 5 | 05/10/2009 |
| Holland | | 39 | 39 | 2 | |
| Hong Kong | 217 | 50 | 267 | 3 | 01/01/2009 |
| India | | 813 | 813 | 7 | |
| Iran | | 51 | 51 | 1 | |
| Israel | 262 | 449 | 711 | 2 | 12/07/2009 |
| Italy | | 158 | 158 | 4 | |
| Japan | | 670 | 670 | 6 | |
| Korea | | 2451 | 2451 | 10 | |
| Kuwait | | 196 | 196 | 1 | |
| Lebanon | | 14 | 14 | 1 | |
| Lithuania | | 69 | 69 | 1 | |
| Malaysia | | 1306 | 1306 | 1 | |
| Mexico | | 456 | 456 | 2 | |
| Norway | | 63 | 63 | 1 | |
| Oman | | 125 | 125 | 1 | |
| Saudi Arabia | 47 | 83 | 130 | 2 | 18/07/2009 |
| Singapore | 70 | 538 | 608 | 12 | 27/04/2009 |

| Country reporting data | Number of treatment courses described in literature (by study enrolment type) | | | Total number of papers published | Date of first patient enrolment in prospective research |
|---|---|---|---|---|---|
| | Prospective | Retrospective | Total | | |
| South Africa | 19 | | 19 | 1 | 17/08/2009 |
| South Korea | | 20 | 20 | 1 | |
| Spain | 2942 | 1156 | 4103 | 16 | 23/04/2009 |
| Sweden | | 108 | 108 | 1 | |
| Switzerland | 78 | 11 | 89 | 2 | 01/10/2009 |
| Taiwan | 75 | 99 | 174 | 3 | 15/07/2009 |
| Thailand | 24 | | 24 | 1 | 08/07/2009 |
| Turkey | 18 | 874 | 892 | 5 | 01/11/2009 |
| UK | 200 | 44 | 244 | 4 | 01/09/2009 |
| USA | 251 | 2308 | 2555 | 25 | 23/04/2009 |
| **Total** | **5278** | **28599** | **33869** | | |

Appendix 5: Country level data demonstrating number of treatment courses described in the literature, and date of first enrolment in prospective research, where relevant. Date in DD/MM/YYYY format.

**Appendix 6 –**Prisma flow diagram describing screening, eligibility and inclusion for clinical trial registration records for studies aimed at enrolling A(H1N1)pdm09 patients.

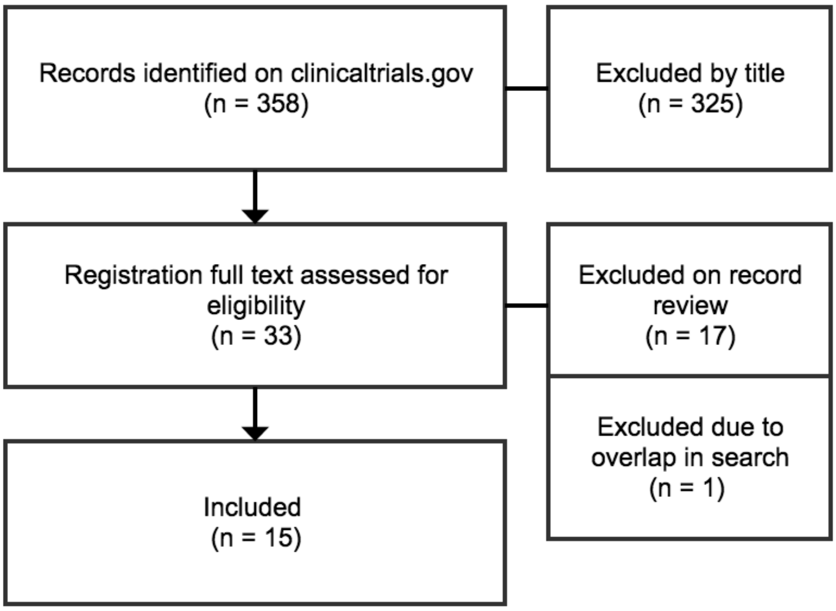

**Appendix 7: Enrolment metrics for studies enrolling A(H1N1)pdm09patients, when analysis is restricted to 'serious' and hospitalized patients.**

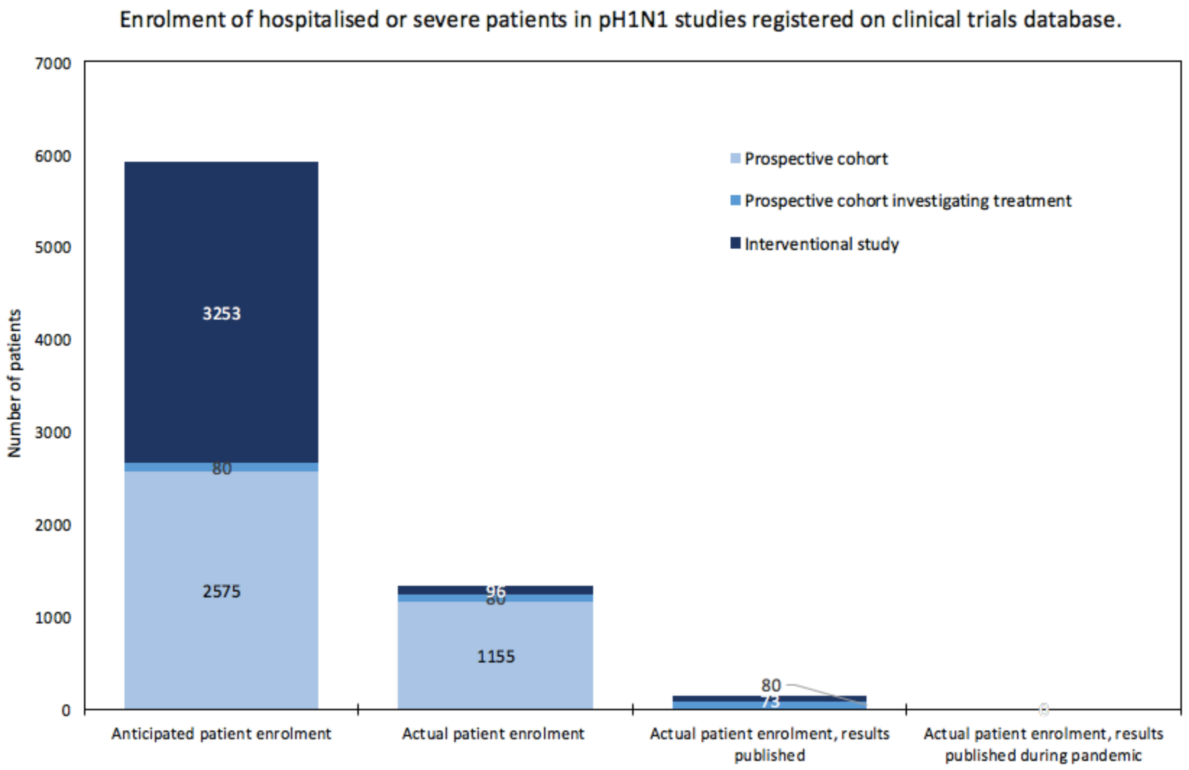


Enrolment metrics for studies enrolling A(H1N1)pdm09patients, when analysis is restricted to 'serious' and hospitalized patients. 10 records are eligible for inclusion. This includes 6 interventional trials and 4 prospective observational studies. Registered trials intended to assess the following treatments: convalescent plasma, IVIG, Chinese medicines, rosuvastatin, and rapamune. Together, the trials intended to enrol 5908 patients, 1331 patients were actually enrolled, results are publicly available for 153 patients, and for no patient were the trial results available before the end of the epidemic. Results are now available for three studies

**Appendix 8: Prisma flow diagram describing screening, eligibility and inclusion for clinical trial registration records for studies aimed at enrolling influenza patients, that were open during the pandemic period.**

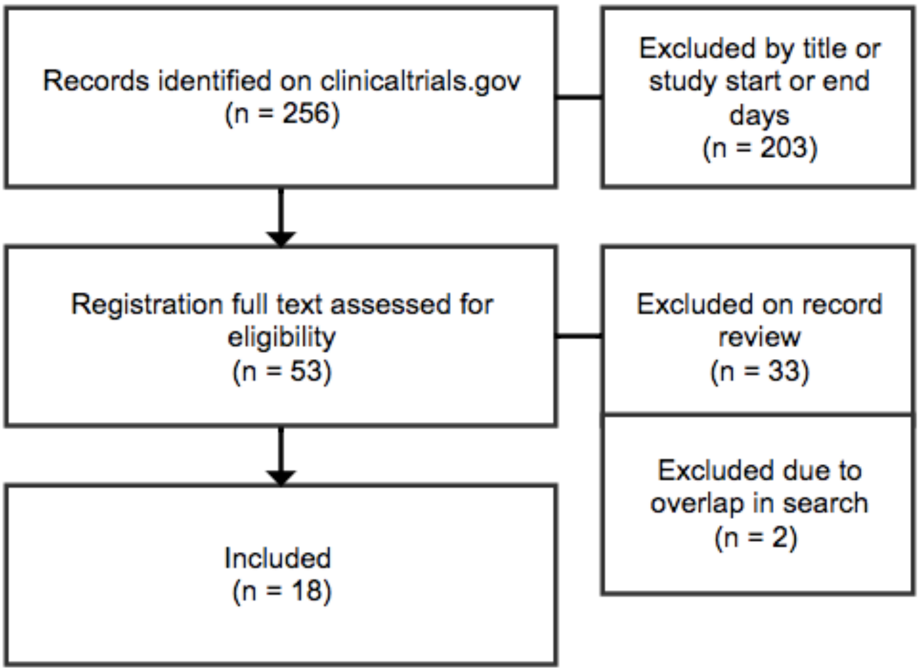

Supplement: Supplementary file 1 — Additional file 1: Appendix 1. Checklist summarising compliance with MOOSE and PRISMA guidelines. Appendix 2. Details of search strategy. Appendix 3. Details of clinical registry search strategy and data extraction. Appendix 4. Studies included in systematic review. Appendix 5. Country level data demonstrating number of treatment courses described in the literature, and date of first enrolment in prospective research, where relevant. Appendix 6. Prisma flow diagram describing screening, eligibility and inclusion for clinical trial registration records for studies aimed at enrolling A(H1N1)pdm09 patients. Appendix 7. Enrolment metrics for studies enrolling A(H1N1)pdm09patients, when analysis is restricted to ‘serious’ and hospitalized patients. Appendix 8. Prisma flow diagram describing screening, eligibility and inclusion for clinical trial registration records for studies aimed at enrolling influenza patients, that were open during the pandemic period. [file 12916_2020_1732_MOESM1_ESM.pdf]
